# 327. Understanding Current Clinical Scenarios for Plasma Microbial Cell-free DNA Diagnostic Testing toward Informing Diagnostic Stewardship

**DOI:** 10.1093/ofid/ofac492.405

**Published:** 2022-12-15

**Authors:** Constance Lau, T Matthew Hill, Kevin Messacar, Frederick S Nolte, Sarah Y Park

**Affiliations:** Karius, Inc., Redwood City, California; Karius, Inc., Redwood City, California; University of Colorado, Children’s Hospital Colorado, Aurora, Colorado; Karius, Inc., Redwood City, California; Karius, Inc., Redwood City, California

## Abstract

**Background:**

Plasma microbial cell-free (mcfDNA) testing has emerged as a promising tool for unbiased detection of pathogens in patients with suspected infections. A systematic evaluation of the patient populations in which this test is used may provide insight into applications for clinical use. We describe demographic and clinical characteristics of patients tested with plasma mcfDNA linked to the Premier Healthcare Database (PHD).

**Methods:**

A retrospective cross-sectional analysis was conducted using the PHD: a US hospital-based, service-level, all-payer database containing information primarily from geographically diverse communities, teaching hospitals, and health systems. Patients with plasma mcfDNA testing 4/1/2018-12/31/2020 were deterministically linked to the PHD. Patient characteristics were collected via chargemaster data, ICD-10 diagnosis, and procedure codes. Immunocompromised (IC) status was identified using 2021 Agency for Healthcare Research (AHRQ) ICD-based definitions. The AHRQ Elixhauser Comorbidity Index score was derived using ICD-10 codes to indicate comorbidity burden.

**Results:**

A total of 778 patients in the PHD underwent plasma mcfDNA testing during the study period, 88% of whom were tested in the inpatient setting (Table 1). Thirteen percent had a discharge diagnosis of sepsis with an unspecified organism. Median inpatient length of stay was 10 days with a median of 4 days from time of admission to plasma mcfDNA testing. Forty-seven percent of patients were IC with a majority (62%) having an Elixhauser score ≥ 7, suggestive of a high comorbidity burden (Table 2). Two hundred forty-nine (32%) inpatients required intensive care unit (ICU) level care for a median 15 days.

**Figure:**
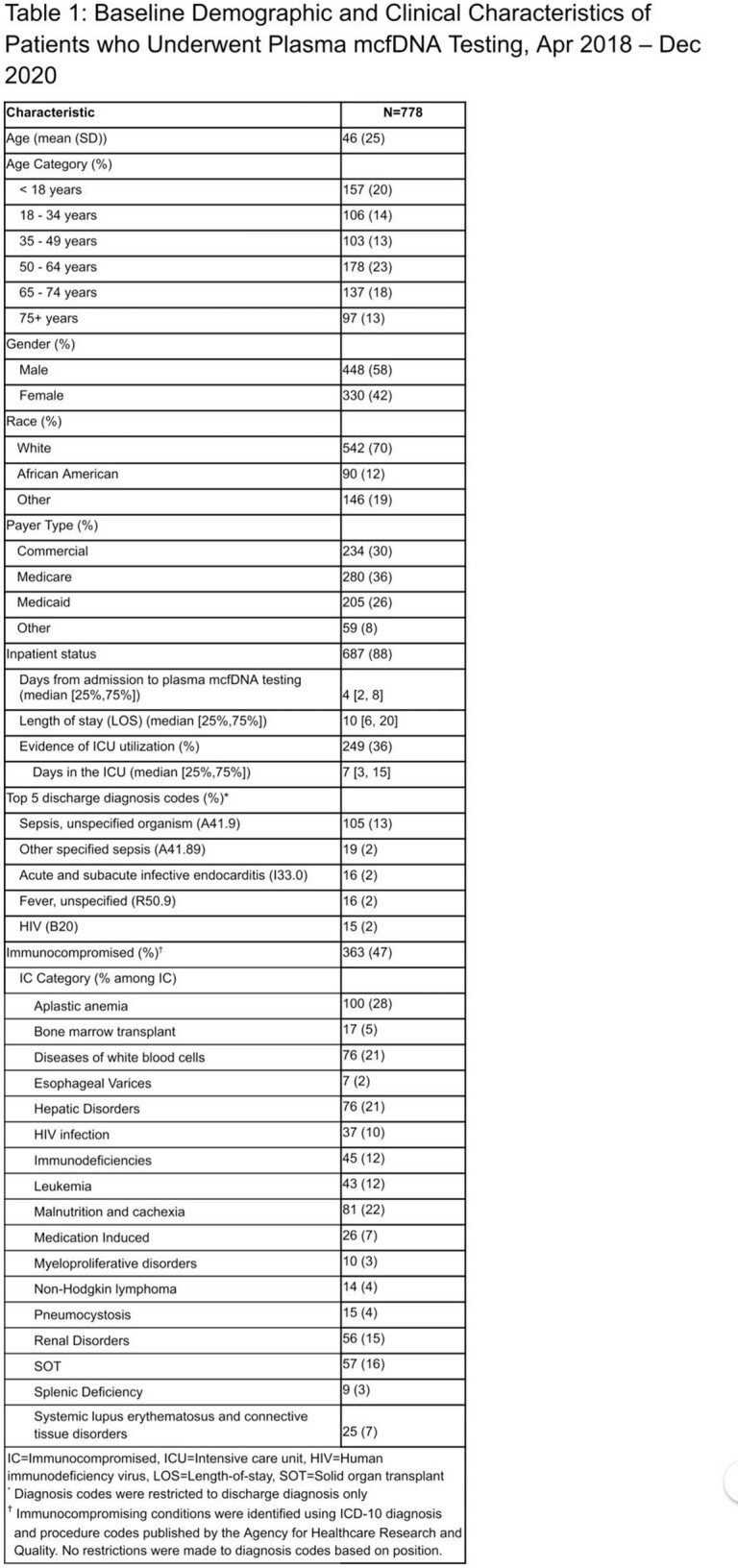

**Figure:**
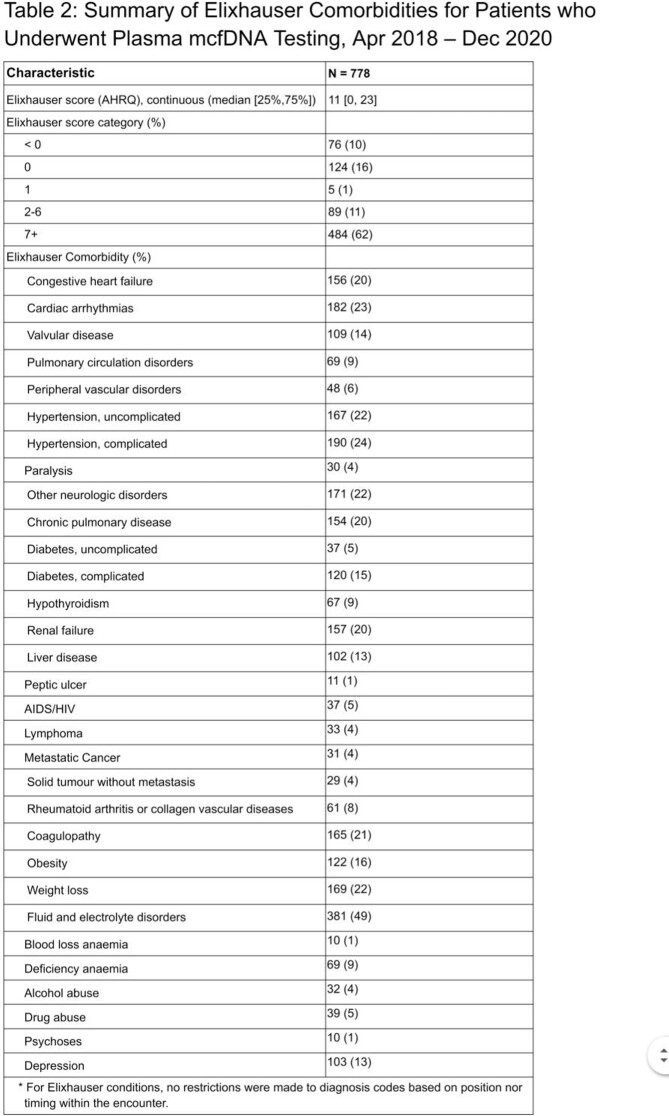

**Conclusion:**

Plasma mcfDNA testing is primarily being used in the inpatient setting in a wide variety of clinical scenarios, particularly among seriously ill and immunocompromised patients, who tend to have broad infectious disease differentials and high morbidity and mortality risks. Understanding the current populations, indications, and timing of mcfDNA testing may contribute to developing diagnostic stewardship research and guidelines to optimize impact on clinical outcomes.

**Disclosures:**

**Constance Lau, MPH**, Karius, Inc.: Current employee|Karius, Inc.: Stocks/Bonds **T. Matthew Hill, PharmD, PhD**, Karius, Inc: Paid employee|Karius, Inc: Stocks/Bonds **Frederick S. Nolte, PhD, D(ABMM), F(AAM)**, Karius: Employee.

